# Enhancing cancer therapy: advanced nanovehicle delivery systems for oridonin

**DOI:** 10.3389/fphar.2024.1476739

**Published:** 2024-12-03

**Authors:** Yilin Su, Lisha Liu, Chongyang Lin, Dashi Deng, Yunfei Li, Mou Huang, Yu Wang, Kangqiu Ling, Haobing Wang, Qiyu Chen, Guixiao Huang

**Affiliations:** ^1^ Institute of Urology, The Third Affiliated Hospital of Shenzhen University, Shenzhen University, Shenzhen, China; ^2^ Institute of Pain, The Affiliated Hospital of Southwest Jiaotong University, The Chengdu Third People’s Hospital, Chengdu, China

**Keywords:** oridonin, drug delivery system, nanoparticle, antitumor, carrier

## Abstract

Oridonin (ORI), an ent-kaurane diterpenoid derived from Rabdosia rubescens (Hemsl.) H.Hara, serves as the primary bioactive component of this plant. It demonstrates a broad spectrum of therapeutic activities, including moderate to potent anticancer properties, alongside anti-inflammatory, antibacterial, antifibrotic, immunomodulatory, and neuromodulatory effects, thus influencing diverse biological processes. However, its clinical potential is significantly constrained by poor aqueous solubility and limited bioavailability. In alignment with the approach of developing drug candidates from natural compounds, various strategies, such as structural modification and nanocarrier systems, have been employed to address these challenges. This review provides an overview of ORI-based nano-delivery systems, emphasizing their potential to improve the clinical applicability of oridonin in oncology. Although some progress has been made in advancing ORI nano-delivery research, it remains insufficient for clinical implementation, necessitating further investigation.

## 1 Introduction

Cancer remains a critical global health concern and continues to rank as the second leading cause of death in the United States (Siegel et al., 2024). In 2020, approximately 19.3 million new cancer cases were reported globally, along with 10 million deaths attributed to cancer (Sung et al., 2021). Conventional treatment modalities, including surgery, chemotherapy, and radiotherapy, remain the cornerstone of cancer management but are often accompanied by severe toxic side effects and the emergence of drug resistance (Bell, 2010). Medicinal plants have long been recognized as a vital source for the discovery of novel anticancer agents (Luo et al., 2019). Notably, over half of the new drugs approved between 1982 and 2002 were either directly or indirectly derived from natural sources. Historically, more than 60% of natural compounds and traditional Chinese herbal medicines have been utilized for their anticancer properties (Tran et al., 2017). Oridonin (ORI), an ent-kaurane diterpenoid extracted from Rabdosia rubescens (Hemsl.) H.Hara, is the principal active compound of this herb, known as Donglingcao in traditional East Asian medicine (Tan et al., 2011). This plant exhibits a range of pharmacological effects, including anti-tumor (Liu et al., 2012; Liu et al., 2021; Li et al., 2023a), anti-inflammatory (Ku and Lin, 2013), anti-microbial (Chen et al., 2023a; Wang et al., 2024), anti-aging (An et al., 2022; Yasuda et al., 2022), anti-fibrotic (Gao et al., 2022), and central nervous system modulatory activities (Liu and Du, 2020; Li et al., 2023b). ORI has garnered increasing attention due to its potent ability to induce apoptosis, inhibit protein synthesis (Li et al., 2016), and exert anti-angiogenic and anti-metastatic effects in both *in vitro* and *in vivo* tumor models (Abdullah et al., 2021; Tian et al., 2017).

Extensive research has demonstrated ORI’s ability to suppress the proliferation of various cancer types, such as breast (Wang et al., 2013; Wu et al., 2016; Yao et al., 2020), gastric (Gao et al., 2023), colorectal (Zhou et al., 2023), thyroid (Liu et al., 2022), and ovarian cancers (Wang and Zhu, 2019). Nevertheless, ORI’s clinical utility is limited by its poor water solubility, low bioavailability, and associated toxicities, including pharmacological and cardiotoxic effects observed in preclinical studies (Li et al., 2021; Xu et al., 2024). Consequently, developing strategies to mitigate these side effects is essential for advancing ORI as a viable anticancer agent (Zhang et al., 2020). Nanoparticles (NPs), as drug carriers, are capable of improving the distribution of drugs in the body, increasing their bioavailability, and enabling targeted delivery to tumor tissues due to their small size and modifiable surfaces. In recent years, nanotechnology has been employed to develop various diagnostic and therapeutic strategies for cancer. Several relatively safe and low-toxicity nanotechnology strategies have demonstrated clinical development potential, including photodynamic therapy (PDT) (Hao et al., 2022; Gunaydin et al., 2021), photothermal therapy (PTT) (Zhao et al., 2021; Qin et al., 2023), and immunotherapy (Barnestein et al., 2022; Li et al., 2022). The focus of drug delivery systems based on nanocarriers lies in enhancing the permeability and retention of drugs or functionalizing the carrier surfaces for targeted delivery (Lv et al., 2024). These systems offer numerous advantages, such as minimizing off-target toxicity, shielding cytotoxic drugs from degradation, extending drug half-life, increasing drug payload and solubility, and reducing renal clearance, making them highly promising for cancer treatment (Zhang et al., 2023a). This review explores various ORI delivery platforms and fabrication techniques, including polymeric nanoparticles, micelles, liposomes, microemulsions, protein NPs, metallic and non-metallic NPs, and cocrystal formations. The review also evaluates the potential of ORI-based drug delivery systems in enhancing bioavailability and specificity, reducing toxicity, and highlights future research directions based on current progress in the field.

## 2 ORI properties

### 2.1 Plant origin and distribution

Isodon rubescens has been utilized in traditional Chinese medicine (TCM) for over a millennium, first documented in the Shen Nong Ben Cao Jing. In the Compendium of Materia Medica, it is categorized as a superior herb, traditionally prescribed for symptoms such as cough, wheezing, phlegm, and chest tightness. The Supplement to Materia Medica also notes its efficacy in treating persistent cough, hemoptysis, and chest pain. In the early 1970s, during a survey of traditional medicine resources in China, researchers discovered that residents of Linzhou City, Henan Province—an area with a high incidence of esophageal cancer—were using a solution derived from Isodon rubescens, which alleviated symptoms of esophageal cancer and pharyngitis. This discovery spurred interest in its anticancer properties, leading to the isolation and identification of its medicinal components, including the extraction of its primary active ingredient, ORI. Isodon rubescens grows predominantly in the Taihang Mountains of Hunan Province, China, flourishing on sun-exposed slopes at altitudes ranging from 100 to 2,800 m (Chen et al., 2022).

**FIGURE 1:**
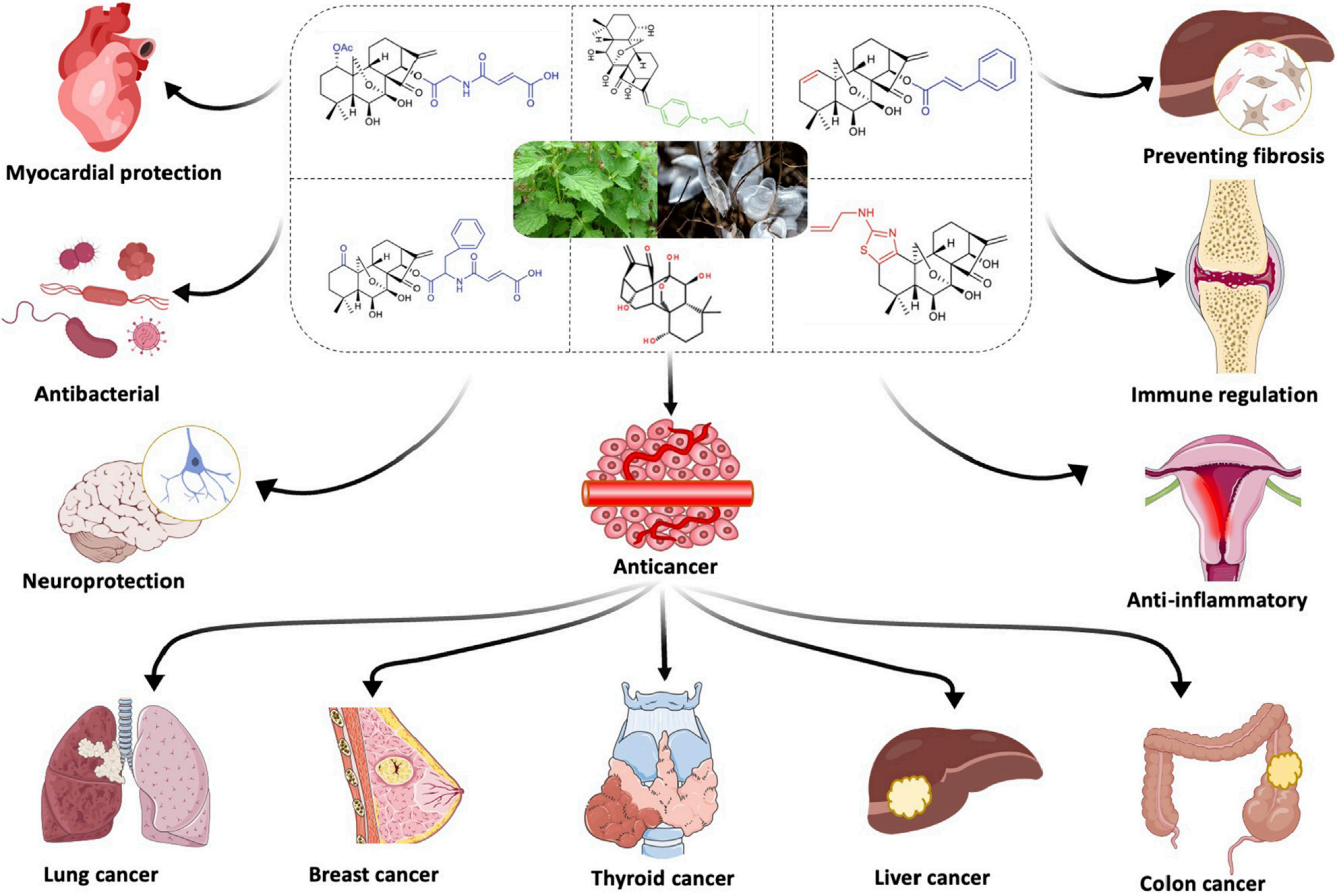
Schematic diagram of ORI derivatives and their efficacy.

Isodon rubescens is classified into four varieties based on the types and concentrations of its primary chemical constituents, ent-kauranoid diterpenoids. The first variety, found in the Taihang Mountains in Jiyuan, Henan Province, mainly contains 7,20-epoxide ent-kauranoid diterpenoid derivatives. The second variety, including *Lu* Rabdosia and *Gui* Rabdosia from Henan Province, predominantly features 6,7-fractured-ent-kaurane diterpenoids and ORI. The third variety consists of Rabdosia rubescensin and Lushan Rubescensin, while the fourth, Xin Rabdosia, primarily contains unoxidized ent-kaurane diterpenoids at the C-20 position. Notably, ORI and ORI B are found exclusively in the Jiyuan variety from Henan Province. ORI’s main chemical structure is the diterpenoid compound, specifically the ent-kaurene tetracyclic diterpenoid represented by its molecular formula C_20_H_28_O_6_ (Sun et al., 2006; Xie et al., 2011) (Figure 1).

### 2.2 Molecular characteristics and anticancer effects of ORI

Various ORI derivatives have shown enhanced efficacy and improved pharmacokinetic properties (Zhang et al., 2020; Sobral et al., 2023). The α-methylene cyclopentanone on ORI’s D-ring plays a critical role in its anticancer function, which can be diminished by ring cleavage or methylene saturation (Sun et al., 1995). Furthermore, the hydrogen bond between the 6-hydroxy and 15-carbonyl groups increases the electrophilicity of C-17, thereby strengthening its interaction with electrophilic enzymes in tumor cells. For example, cytotoxicity and water solubility have been improved by adding hydrophilic side chains to the 1-O- and 14-O-hydroxyl groups (Xu et al., 2016). Modifying the A-ring of ORI A, such as by introducing new functional groups, has been shown to enhance apoptosis induction (Ding et al., 2013). The D-ring enone of dong quinone is also essential for its anticancer effects, and derivatives with substituted benzene moieties at the C-17 position have demonstrated improved apoptotic induction and G2 phase arrest (Shen et al., 2018) (Figure 1).

Despite the well-documented anticancer potential of ORI, its clinical application is hindered by rapid blood concentration decline following intravenous administration, poor water solubility, and oral bioavailability of less than 5% (Xu et al., 2006). Advances in nanotechnology have led to the development of multifunctional nanoplatforms designed to improve ORI’s bioavailability, holding promise for clinical use (Ali et al., 2024). However, while ORI derivatives have been widely investigated in cancer therapy, a comprehensive review focusing on the anticancer advantages and mechanisms of ORI-based nanoparticle drug delivery systems (DDS) is still lacking.

### 2.3 Anticancer mechanisms of ORI

**FIGURE 2:**
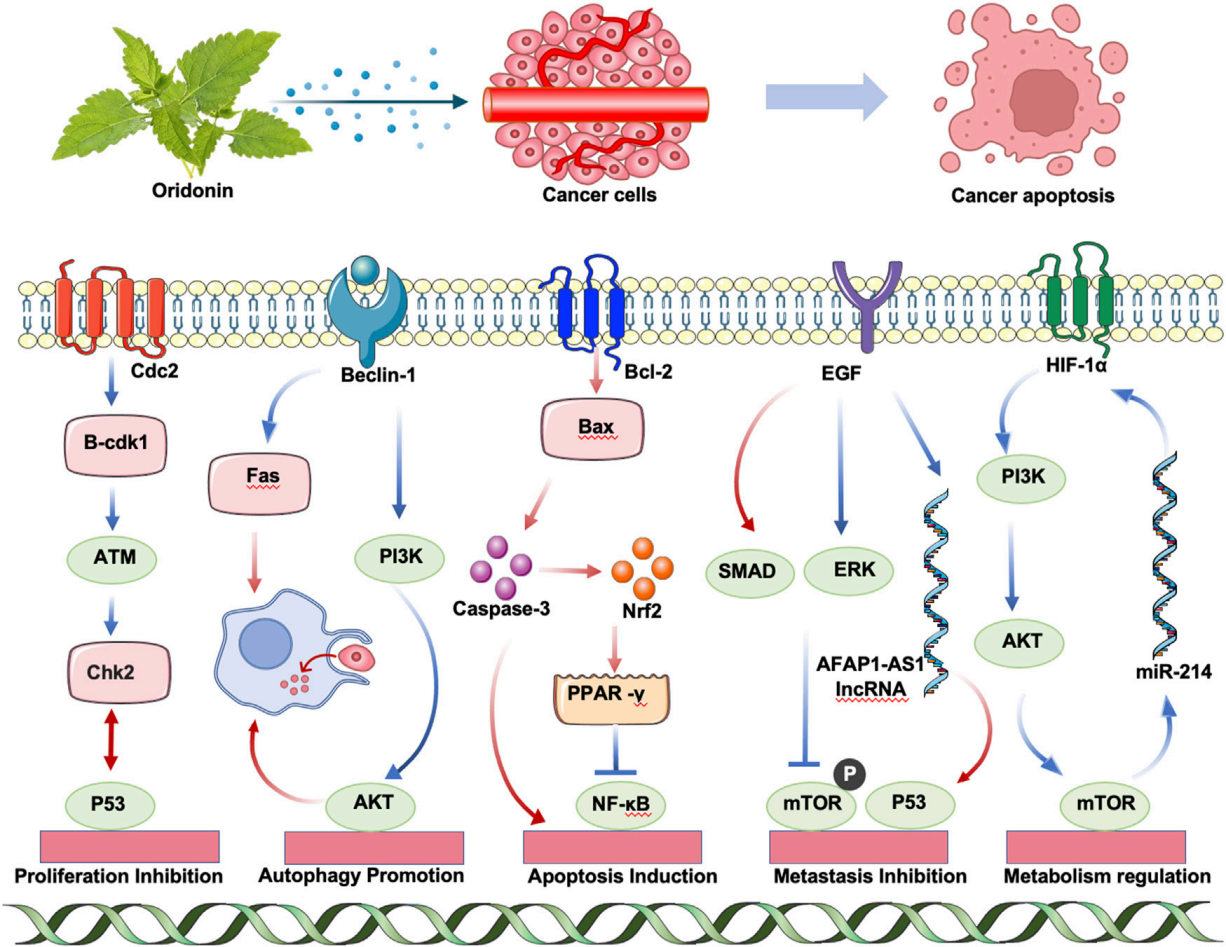
Schematic representation of ORI’s anti-tumor mechanisms.

Decades of research have revealed that ORI exerts anticancer effects through multiple mechanisms, including inducing tumor cell apoptosis and autophagy, inhibiting proliferation, preventing angiogenesis and metastasis, and enhancing radiosensitivity (Liu et al., 2012; Li et al., 2011a). Recently, ORI has gained recognition as a promising anticancer agent due to its multifaceted activity across various malignancies via distinct pathways (Figure 2).

#### 2.3.1 Proliferation inhibition

Cdc2, a key regulator of mitosis in fission yeast, functions as a protein kinase activated through cyclin binding, thereby driving cell cycle progression (Suski et al., 2021). Cyclin B-CDK1, critical for controlling events associated with cell cycle regulation, predominantly governs mitotic progression and cell division, leading to G2/M phase arrest (Bury et al., 2021). Furthermore, ataxia telangiectasia mutated enhances G2/M arrest in A549 cells (Zheng et al., 2017). Recent studies suggest ORI induces G2/M arrest through these pathways (Qi et al., 2012). In addition, ORI inhibits HepG2 proliferation in a time- and dose-dependent manner, arresting the cell cycle at the S phase (Li et al., 2020a).

#### 2.3.2 Autophagy promotion

Autophagy, a vital metabolic process, involves the degradation and recycling of cellular components through lysosomal delivery. Research shows that low doses of active ORI enhance the phagocytosis of apoptotic cells by macrophage-like U937 cells and induce autophagy in these cells (Zang et al., 2012). ORI also promotes macrophage phagocytosis of apoptotic bodies via the autophagy-lysosomal pathway, mediated by toll-like receptor (TLR)4 signaling, enhancing cell migration (Zang et al., 2019). Additionally, ORI triggers apoptosis through the extrinsic apoptotic pathway involving Fas and FasL signaling cascades (Zang et al., 2013).

#### 2.3.3 Apoptosis induction

ORI induces apoptosis or necrosis of tumor cells in a dose-dependent manner by modulating several pathways linked to apoptosis, including the mitogen-activated protein kinase (MAPK) family and phosphoinositide 3-kinase (PI3K)/AKT pathways. In breast cancer cells, ORI enhances apoptosis by promoting autophagy. Furthermore, it increases the radiosensitivity of lung cancer cells by upregulating Bax and downregulating Bcl-2, facilitating radiation-induced cell death by accelerating DNA damage in non-small-cell lung cancer (Li et al., 2018a).

#### 2.3.4 Metastasis inhibition

ORI also inhibits cancer invasion and metastasis by suppressing the expression of VEGF family members such as ascular endothelial growth factor (VEGF)-A, VEGFR-2, and VEGFR-3 (Jiang et al., 2020). It impedes metastasis through mechanisms such as the inhibition of the TGF-β/SMAD signaling pathway, blockade of the EGF/epidermal growth factor receptor (EGFR)/extracellular signal-regulated kinase (ERK) signaling cascade (Bu et al., 2019; Che et al., 2021), reduction of mechanistic target of rapamycin (mTOR) signaling pathway phosphorylation, downregulation of lncRNA AFAP1-AS1, and enhancement of p53 expression (Ye et al., 2012; Li et al., 2007). These actions collectively underscore ORI’s potential as an effective therapeutic agent against cancer invasion and metastasis.

#### 2.3.5 Metabolism regulation

The aerobic glycolysis characteristic of tumors, known as the Warburg effect, is closely linked to the overexpression of hypoxia-inducible factor 1 alpha (HIF-1α), a pivotal regulator of tumor energy metabolism (Chelakkot et al., 2023). Research has shown that ORI suppresses glycolytic flux by reducing HIF-1α expression and concurrently lowering vascular endothelial factor levels, thereby inhibiting angiogenesis in breast cancer cells such as MDA-MB-231 and 4T1 (Liu et al., 2024a). This ultimately results in the inhibition of breast cancer cell proliferation. Additionally, studies indicate that ORI upregulates miR-214 expression in H9c2 rat cardiomyocytes, affecting the PI3K/AKT/mTOR signaling pathway, which subsequently downregulates HIF-1α expression and inhibits glycolytic activity. This process induces apoptosis and autophagy in H9c2 cells under hypoxic conditions.

#### 2.3.6 Mechanism of action of ORI in different cancers

Previous research has extensively explored ORI’s effects across various cancer cell lines, including colorectal, breast, lung, gastric, and esophageal cancers, by investigating its mechanisms and pathways of action. The inhibition mechanisms of ORI across different cancers are detailed in Table 1.

**TABLE 1:** Anticancer activities of ORI in various cancers.

Cancer	Cell types	Outcomes	Mechanisms	Refs
Colorectal cancer	LSCC	Promoting cancer cell death	Activated TP53 and inhibited TCF4 transactivation, which aggravated the increase of ROS level and calcium release in tumor cells	Zhou et al. (2023)
	CRC	Promoting apoptosis of cancer cells	Activation of PKM2 and inhibition of glycolysis	Chen et al. (2023b)
	SW1116 HT29 HCT116	Induced cell autophagy	Activation of p21 and p27 and p16 expression and cut c-myc gene expression	Gao et al. (2010)
	SW1116	Induction of apoptosis and senescence in colon cancer cells *in vitro* and *in vivo*	Increased hydrogen peroxide and glutathione in colorectal cancer cells	Gao et al. (2012)
	SW480	Inhibition of proliferation, induction of apoptosis	Glucose uptake was inhibited, and the protein levels of GLUT1 and MCT1 were downregulated to reduce lactate output to induce metabolic imbalance	Yao et al. (2017)
Breast cancer	4T1 MCF-7 MDAMB-231	Inhibition of cell proliferation	Down-regulating the expression of important proteins in PI3K/AKT/mTOR signaling pathway	Zhang et al. (2023b)
	4T1	Promote cell apoptosis	Inhibition of Notch 1-4 expression in the Notch pathway	Xia et al. (2017)
	HMEC-PIK3CA H1047R	Inhibition of tumor cell growth	Blocking AKT-mTOR signaling	Sun et al. (2018)
	MDA-MB-231	Inhibited of migration, invasion, adhesion and angiogenesis	Angiogenesis via downregulation of the HIF-1α/VEGF signaling pathway	Li et al. (2018b)
Gastric cancer	SGC-7901	Inhibition of proliferation Induction of apoptosis	Regulates the TNF-α/AR/TGF-β signaling pathway axis	Gao et al. (2023)
	SGC-7901 HGC-27	Induction of migration	Reduce Bcl-2/Bax ratio, leading to mitochondrial dysfunction, causes cytochrome c release and activates caspase-3	Gao et al. (2016) and Sun et al. (2012)
	SNU-216	Promote cell apoptosis	Significantly upregulated the mRNA and protein expression of p53	Bi et al. (2018)
Lung cancer	H1688 BEAS-2B HBE	Targeted inhibition of migration	Increases E-cadherin expression to inhibit EMT	Xu et al. (2020)
	A549	Promotion NK cell immune effect	The expression of CD107a and IFN-γ was upregulated	Hwang and Chang (2023)
Esophagus cancer	KYSE-30 KYSE-150 EC9706	Induction of cell apoptosis	Inhibition of PI3K/AKT/mTOR and Ras/Raf pathways	Jiang et al. (2019)
Pancreatic cancer	SW1990	Apoptosis and inflammation	JNK and p38 pathways, MAPK, p53	Bu et al. (2014)
Bladder cancer	UMUC3 T24	Inhibits of proliferation, promotes apoptosis, antagonizes immune evasion	Inhibit for HK1	Liu et al. (2024a)
Myelogenous Leukemia	OCIM2 OCI-AML3	Promote cell apoptosis	Significantly upregulated the p53 and p14 arf protein	Hu et al. (2020)
Leukemia	K562 HL60	Promotes the degradation of oncogenic proteins	Oridonin shorted the half-life of the BCR-ABL protein	Huang et al. (2017)
	K562-R882H K562-tdTomato	Induction of cell apoptosis	Activation of the RIPK1-Caspase-8-Caspase-3 and RIPK1-RIPK3-MLKL pathways	Liao et al. (2021)

## 3 Current status of bibliometric analysis of DDS for ORI

**FIGURE 3:**
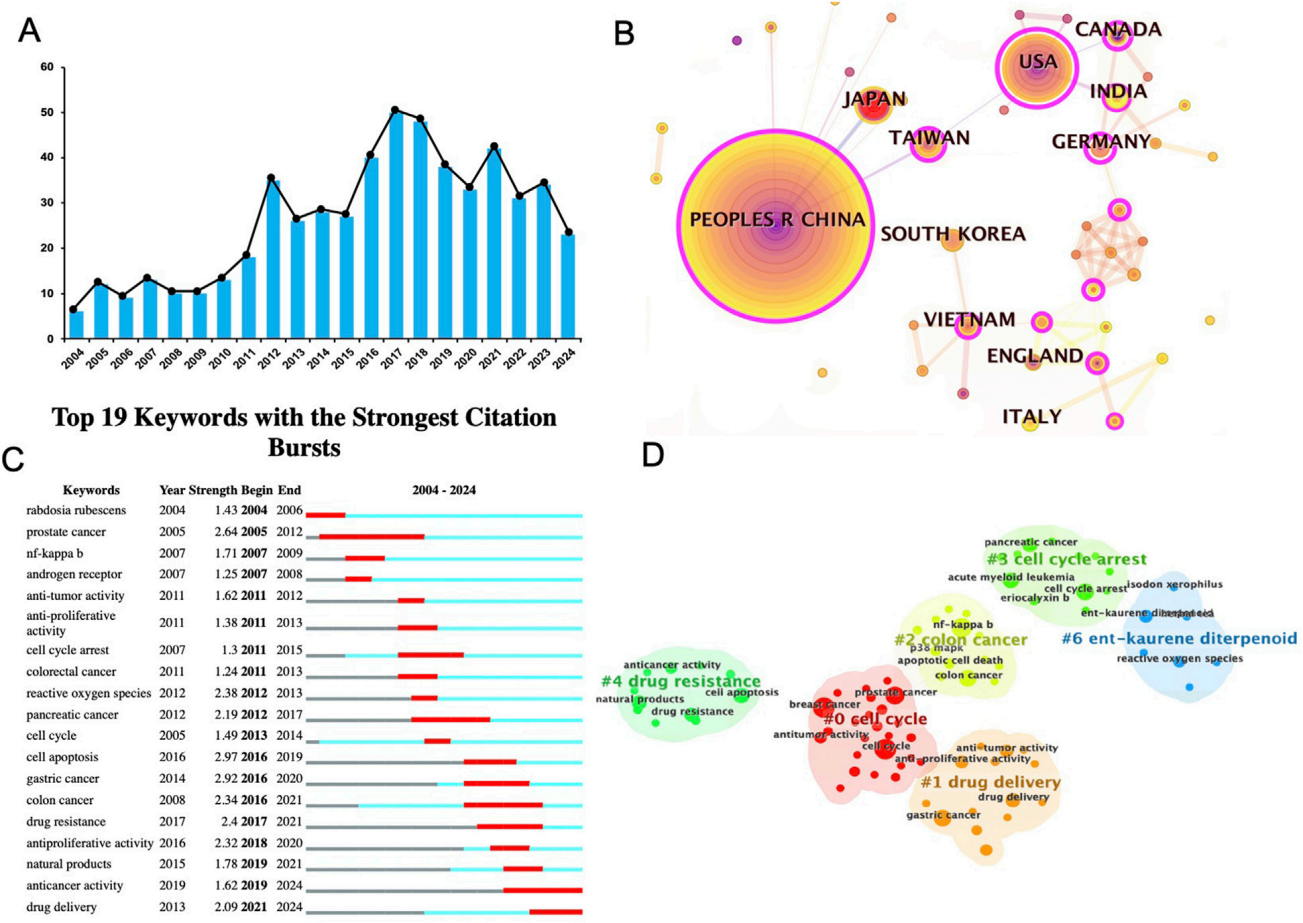
Current status of bibliometric analysis of DDS for ORI. **(A)** Annual publication trends (2004–2024); **(B)** Global research distribution; **(C)** Top keywords with the strongest citation bursts (2004–2024); **(D)** Keyword clusters in Rabdosia rubescens research.

To assess the current landscape of drug delivery research on Rabdosia rubescens meglumine, a comprehensive analysis of keywords and study distribution was conducted. An extensive review of publications related to Rabdosia rubescens in cancer treatment from 2004 to 2024 was performed using search engines such as Web of Science and PubMed. Figure 3A reveals a general upward trend in research publications, with peaks in 2016, 2018, and 2020, followed by a notable decline in 2023 and a slight recovery in 2024.

Additionally, a global research distribution map (Figure 3B) was developed to summarize the countries contributing to studies on Rabdosia rubescens meglumine for cancer therapy. China emerged as the leading contributor, followed by the United States, Japan, and Germany. Other notable contributors included South Korea, India, Canada, and several European nations, such as the United Kingdom and Italy. This distribution reflects the global interest in leveraging *Rabdosia rubescens* to advance cancer treatment, particularly in drug delivery systems.

Figure 3C highlights the top 19 keywords linked to Rabdosia rubescens research that experienced significant citation bursts, signaling periods of heightened focus on these topics. Keywords such as “drug resistance,” “drug delivery,” and “antiproliferative activity” showed particularly strong bursts, reflecting critical areas of study across various years and emphasizing the clinical significance and potential of ongoing ORI-DDS research.

A brief overview of ORI’s anticancer mechanisms was also conducted, alongside an extraction of keyword clusters related to *R. rubescens* research, as depicted in Figure 3D. This keyword co-occurrence map reveals major clusters focused on key topics such as Cell Cycle (#0), Drug Delivery (#1), Colon Cancer (#2), Cell Cycle Blockade (#3), Drug Resistance (#4), and Ent-Kaurene Diterpenoid (#6). These clusters illustrate key research areas, including mechanisms of action (e.g., cell cycle arrest), therapeutic applications (e.g., drug delivery), and specific cancer types (e.g., colon cancer).

The “Drug Delivery” panel is particularly relevant to this review, as it emphasizes the various technologies developed to optimize cynarin delivery. Innovations such as nanodelivery systems, liposomes, and other advanced DDS have been designed to enhance the therapeutic efficacy of Rabdosia rubescens while minimizing potential side effects.

Overall, this analysis provides a thorough examination of *R. rubescens* research in cancer therapeutics, focusing particularly on drug delivery systems. By capturing trends, highlighting key contributors, and identifying significant research topics, the analysis underscores the importance of innovative drug delivery approaches in improving the efficacy of Rabdosia rubescens for cancer treatment.

## 4 Nano drug delivery system with ORI as a functional molecule

Studies indicate that ORI shows significant potential as an anticancer agent. However, its clinical application is hindered by poor water solubility and low bioavailability, which stands at only 5%. Although ORI is generally regarded as safe, recent research has revealed a certain level of cytotoxicity to liver tissue, possibly linked to the inhibition of embryonic development. Additionally, exposure to oridonin (ORI) at concentrations of 25 μM (μM) or higher for 48 h notably elevates cytosolic Ca^2+^ concentration, promotes ceramide formation, and induces erythrocyte death through suicidal pathways (Jilani et al., 2011).

**FIGURE 4:**
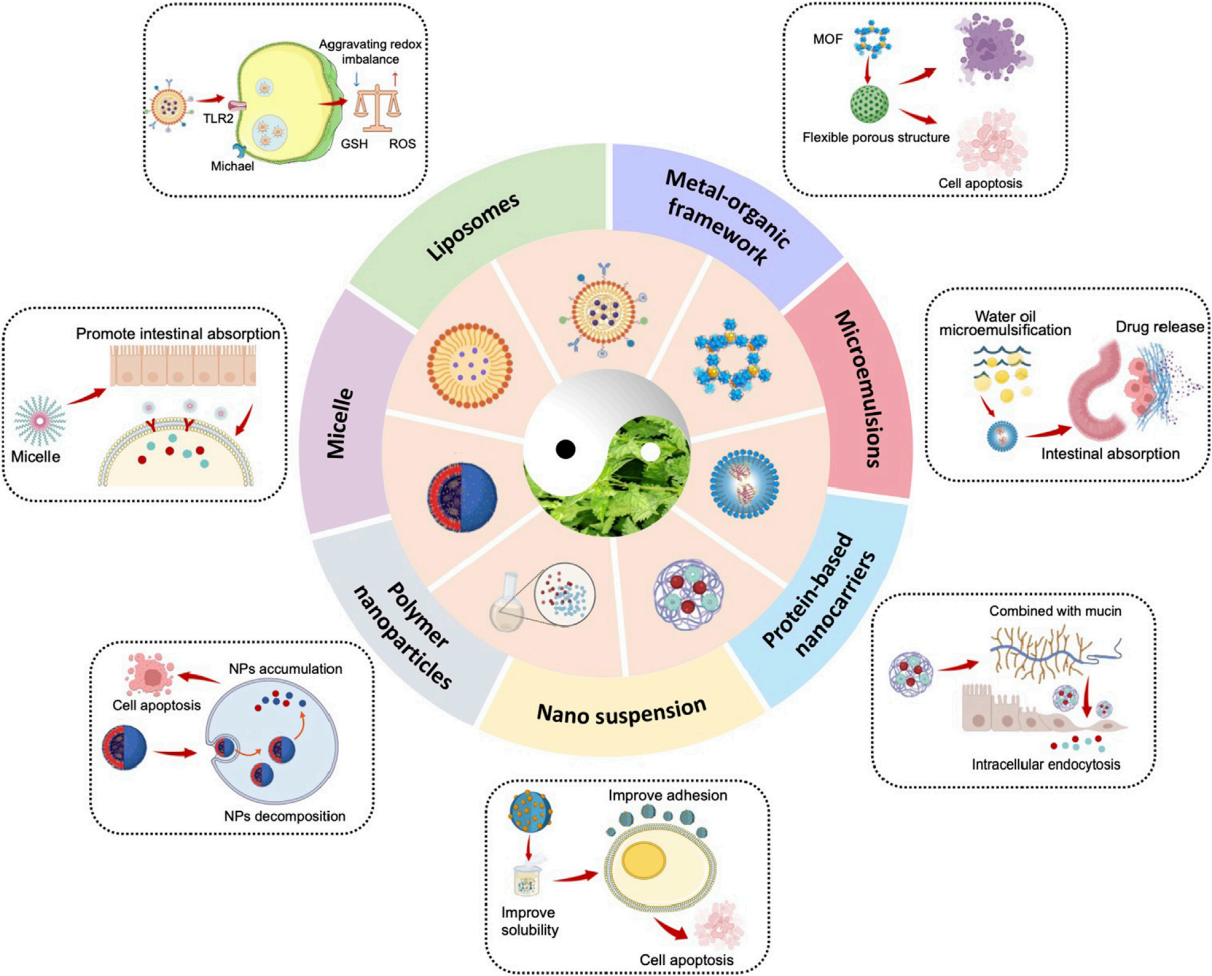
Strategies for ORI nano DDS in cancer therapy.

To address these challenges, the development of advanced drug delivery systems is essential to improve ORI’s solubility, enhance bioavailability, and reduce toxicity (Table 2; Figure 4). Several novel delivery platforms, including liposomes, microemulsions, micelles, protein-based nanocarriers, metal-organic frameworks (MOFs), and nanosuspensions, have been investigated to boost bioavailability, prolong systemic circulation, and mitigate the adverse effects of ORI (Lu et al., 2016).

**TABLE 2:** Summary of ORI DDS in cancer therapy.

Formulations	Preparation method	Advantages	Administration	Refs
Liposome	Ethanol injection method	Enhanced stability and *in vitro* release rate	Intravenous injection	Gao et al. (2021)
Liposome	Lipid injection method	Increased elimination half-life and the area under the concentration-time curve	Intravenous injection	Lin et al. (2023)
Liposome	High-pressure homogenization method	Enhance the ability of drug release and induce apoptosis	Intravenous injection	Chen et al. (2024a)
Micelle	Aqueous solution self-assembly method	Extend the blood circulation time of ORI	Intravenous injection	Xu et al. (2021)
Micelle	Ethanol solvent evaporation method	Improve water solubility and promote intestinal absorption	Oral administration	Ke et al. (2017)
Metal-organic framework (MOF)	Solvent adsorption method	High drug loading and good sustained-release characteristics	*In vitro* drug delivery	Chen et al. (2019)
Metal-organic framework (MOF)	Ultrasonic mixing method	Improve tumor microenvironment and enhance ferroptosis therapy	Intravenous injection	Cai et al. (2024)
Microemulsions	Vortex mixing method	Improve water solubility and bioavailability	Oral administration	Zhang et al. (2008)
Nano suspension	High-pressure homogenization method	Delayed blood clearance and well-targeting	Intravenous injection	Gao et al. (2007)
Nano suspension	Wet medium grinding technology	Improved solubility and permeability	Oral administration	Teran-Saavedra et al. (2019) and Zhang et al. (2016)
Nano suspension	High-pressure homogenization method	Enhanced cytotoxicity *in vitro* and apoptosis-inducing ability *in vivo*	Intravenous injection	Lou et al. (2009)
Lactosylated albumin NPs	Desolvation system preparation method	Targeting of hepatocellular carcinoma	*In vitro* cell administration	Teran-Saavedra et al. (2019)
Galactosylated bovine serum albumin NPs	Desolvation gas technology	Liver tumor targeting vector	*In vitro* drug release	Li et al. (2013)
Protein-based nanocarriers	High-pressure homogenization method	Increased bioavailability	Oral administration	Liu et al. (2017)
Polymer NPs	Interfacial deposition method	Prolong the survival time of mice and improve efficacy	Intravenous injection	Feng et al. (2008)

### 4.1 Liposomes

The clinical advancement of ORI in cancer therapy remains restricted due to its low bioavailability and rapid plasma clearance. Liposomes, a well-established nanocarrier, present significant advantages such as high biocompatibility, low toxicity, and improved drug bioavailability (Wang et al., 2021). They can be engineered for tumor-specific drug delivery, enabling precise targeting of active compounds to tumor cells (Li et al., 2020b). Structurally, liposomes are spherical vesicles comprising one or more phospholipid bilayers, capable of encapsulating both amphiphilic and lipophilic molecules within their matrix (Shah et al., 2020).

**FIGURE 5:**
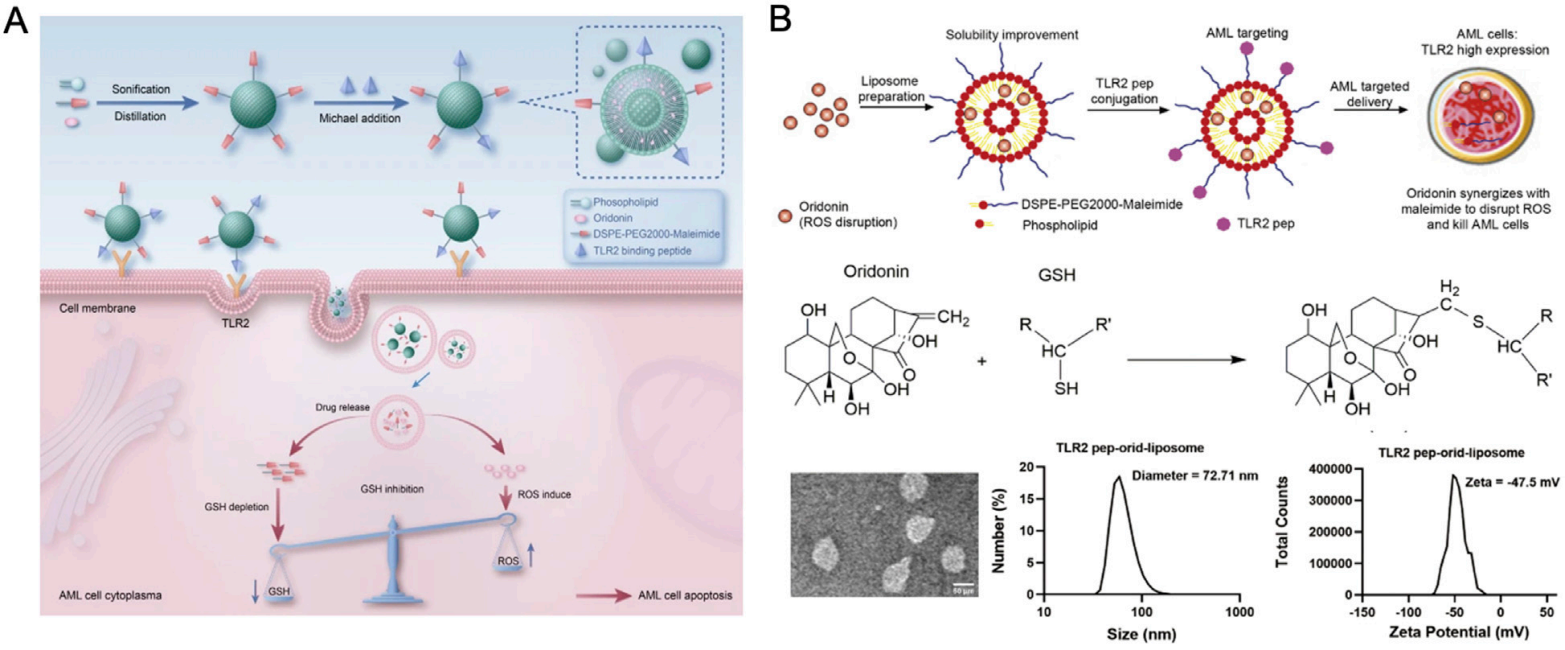
**(A)** Schematic of TLR2 pep-ORI-liposome preparation. **(B)** Material characterization of TLR2 pep-ORI-liposome. Reproduced with permission from Ref (Liu et al., 2024b). Copyright 2024, Springer Nature.

ORI has shown the capacity to disrupt reactive oxygen species (ROS) through covalent interactions with glutathione. Aleimide-liposomes, noted for their efficiency in ROS-targeting delivery systems, have enhanced ORI’s solubility and pharmacokinetic profile, amplifying its ROS-disruptive capabilities. Additionally, ORI-loaded liposomes targeting acute myeloid leukemia (AML) facilitated intracellular glutathione (GSH) depletion, increasing the concentration and specificity of ORI within AML cells (Figure 5) (Liu et al., 2024b). Compared to control groups, ORI liposomes significantly extended the survival of tumor-bearing mice, exhibiting stronger cytotoxic effects and higher safety margins.

Studies have further demonstrated that ethanol-optimized ORI liposomes enter cells via endocytosis, leading to enhanced cellular uptake and accumulation in targeted cells. The strong affinity of liposomes for cell membranes allows ORI encapsulated within liposomes to penetrate cells more efficiently, thereby enhancing its antitumor effects (Wang et al., 2022). Zheng et al. developed a nanostructured lipid carrier (NLC) loaded with ORI, which exhibited higher area under the curve (AUC) values and prolonged bloodstream retention compared to ORI solution, underscoring the potential of NLCs to mitigate ORI’s rapid plasma clearance (Zheng et al., 2012a).

Wang et al. (2009) utilized polyethylene glycol-distearyl phosphatidylethanolamine (PEG2000-DSPE) as a surface coating to formulate stealth liposomes loaded with ORI, which demonstrated significant efficacy in inhibiting solid tumor growth. These liposomes extended the circulation time of ORI in mice, decreased its accumulation in the reticuloendothelial system, and enhanced its anticancer activity. Additionally, ORI-loaded liposome microbubbles targeting the folate receptor exhibited a higher binding affinity for HepG-2 cells, leading to significantly increased anticancer potency (Wang et al., 2017).

Wang et al. (2019) further developed an innovative drug delivery system by covalently linking ORI-containing liposomal microbubbles (LUMO) with folic acid-coupled heme-loaded multi-walled carbon nanotubes (FMTP-LUMO). This dual-targeting platform improved the therapeutic effect in liver cancer, particularly when combined with chemo-sonodynamic therapy, where the tumor inhibition rate surpassed 90%, significantly higher than that of FMTP (42.8%) and LUMO (32.5%) alone (Wang et al., 2019).

Recent studies suggest that anisodamine-calcium lipid phosphate NPs (AS-LCPS) hold promise as an effective carrier for ORI, showing excellent stability and sustained release properties in lung cancer cells. *In vivo* experiments revealed that surface modifications on AS-ORI LCPs enhanced cellular endocytosis, leading to increased intracellular drug accumulation (Shen and Ma, 2022).

Traditional liposomes often lack tumor-specific targeting and sustained-release capabilities, which can limit their ability to significantly improve bioavailability. To address this, researchers have developed various modified liposome formulations. Ethanol and polyethylene glycol-optimized liposomes, for instance, have shown improved tumor cell affinity and enhanced anticancer activity for ORI (Ren et al., 2021). Moreover, complex liposomes targeting tumor cells have been designed to further increase bioavailability, enhance anticancer efficacy, reduce off-target effects, and induce persistent immune memory while curbing metastasis (Lin et al., 2023; Xin et al., 2023a). Combining liposome carriers with sonodynamic and thermal therapies has also yielded promising anticancer results (Xin et al., 2023b).

### 4.2 Micelle

Micelles, synthesized from amphiphilic surfactants in aqueous environments, possess the remarkable ability to self-assemble into nanoscale structures, a key property that makes them ideal drug delivery vehicles (Ghosh and Biswas, 2021). When surfactant concentrations surpass the critical micelle concentration, these molecules organize into nanostructures known as micelles, which are effective carriers for hydrophobic drugs with low water solubility, such as ORI (Del Regno et al., 2021; Guerrero-Hernández et al., 2022).

**FIGURE 6:**
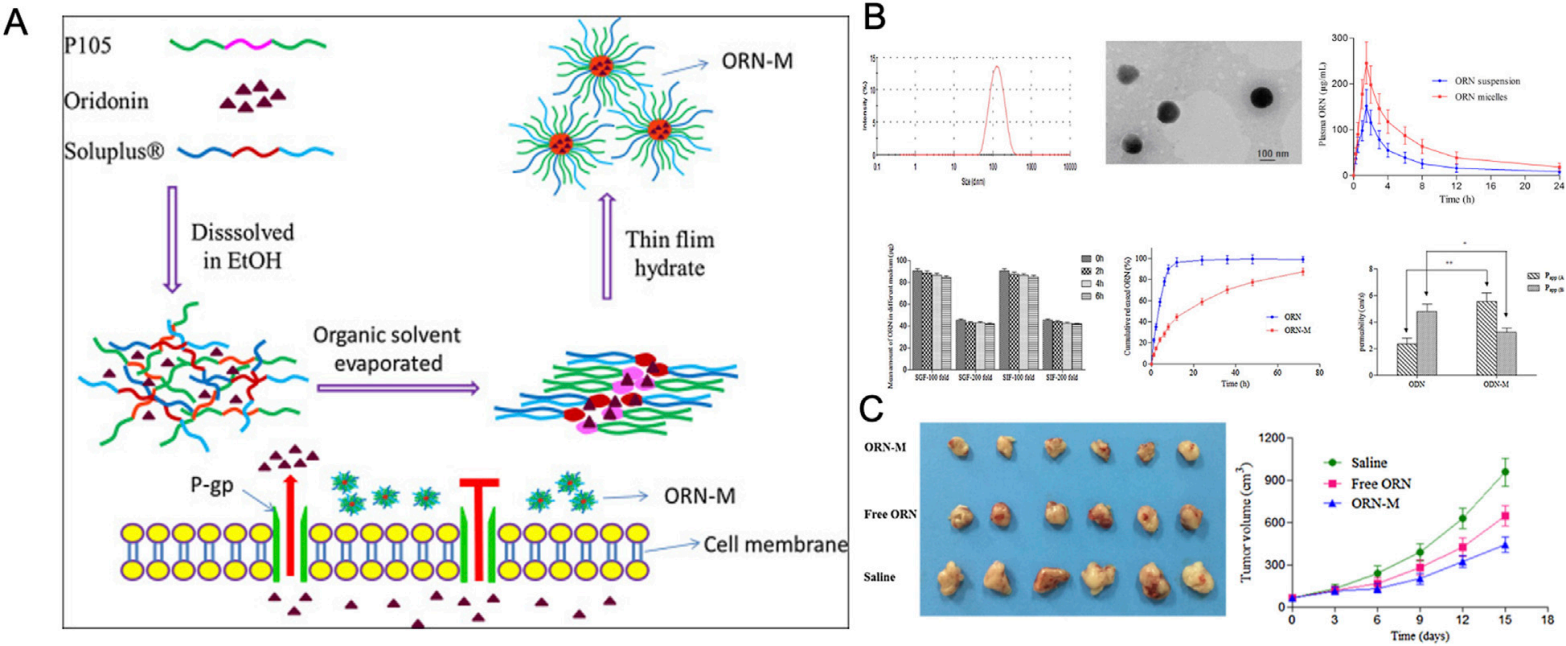
**(A)** Schematic illustration of ORN-loaded mixed micelles. **(B)** Characterization of ORN-suspension and ORN-M materials. **(C)** Tumor inhibition effects after treatment in each group. Reproduced with permission from Ref (Ke et al., 2017). Copyright 2016, Elsevier.

Research indicates that oral drug delivery can improve patient compliance compared to injectable methods. Among various oral preparation techniques, hybrid micelle systems have emerged as one of the most effective for enhancing drug delivery. Due to their core-shell structure, these systems significantly increase the solubility of hydrophobic drugs (Chen et al., 2015). For instance, a nanohybrid micellar system, ORN-M, consisting of Soluplus^®^ (SOL) and Pluronic P105, was developed for the oral delivery of ORI. ORN-M exhibited a long-acting, sustained-release profile *in vitro*, showing stronger inhibition of tumor cell growth. Its anticancer efficacy was further enhanced by improved *in vivo* solubility, permeability, and greater resistance to drug efflux (Ke et al., 2017) (Figure 6).

In another study, monomethoxy poly (ethylene glycol)-poly (epsilon-caprolactone) (MPEG-PCL) was employed as a drug carrier to enhance ORI’s solubility. ORI-loaded MPEG-PCL micelles, prepared using the thin-film hydration method, successfully encapsulated ORI while preserving its anticancer properties. These micelles released ORI in a sustained manner *in vitro*, prolonging its therapeutic action (Xue et al., 2012).

Targeting hepatocellular carcinoma (HCC), Fang et al. designed a hybrid micelle system modified by the peptide Ala-Pro-Asp-Thr-Lys-Thr-Gln (APDTKTQ), aimed at the receptor for advanced glycation end products (RAGE), which is overexpressed in HCC. The ORI-loaded micelles showed improved cellular uptake and induced more apoptosis compared to free ORI micelles, with a uniform spherical shape and no aggregation (Fang et al., 2016).

Xu et al. (2021) synthesized redox-sensitive ORI polymer prodrug formulations, which self-assembled into micelles (P-ss-ORI) via covalent linkage of ORI to polyethylene glycol block polylysine with a disulfide linker. These micelles exhibited small size and high drug-loading efficiency. In gastric cancer treatment, under conditions of high intracellular GSH and low pH, P-ss-ORI demonstrated prolonged ORI retention, tumor tissue accumulation, efficient endocytosis by cancer cells, and rapid, complete drug release (Xu et al., 2021).

Another innovative formulation involved coupling D-α-tocopherol polyethylene glycol succinate (TPGS) with poly (lactic-co-glycolic acid) (PLGA) via a disulfide linker (TPGS-S-S-PLGA) to form ORI micelles. In HCC cells, this design increased cellular uptake and apoptosis induction (Fang et al., 2016). These studies collectively highlight the potential of micelles and their modified versions to enhance anticancer activity, *in vivo* solubility, permeability, and resistance to drug efflux.

### 4.3 Metal-organic framework (MOF)

MOFs represent an emerging class of hybrid porous materials, composed of metal ions or clusters connected by organic linkers (Giliopoulos et al., 2020), which offer significant potential as drug delivery carriers due to their highly tunable structure, pore size, versatile functionality, and improved biocompatibility (Wu and Yang, 2017).

**FIGURE 7:**
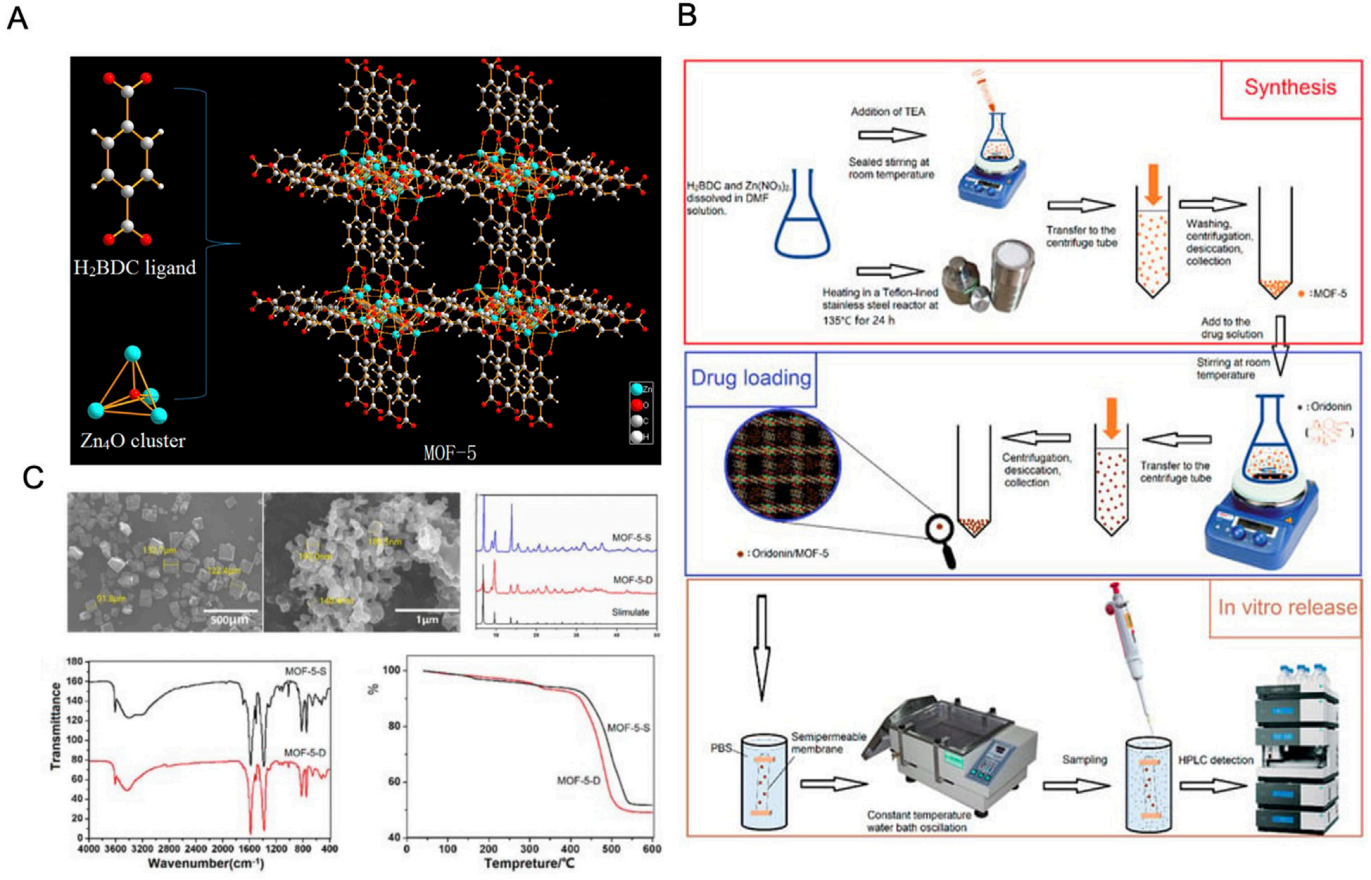
**(A)** Schematic illustration of the MOF-5 construction. **(B)** Schematic diagram of the MOF-5 preparation process. **(C)** Characterization of the MOF-5. Reproduced with permission from Ref (Chen et al., 2019). Copyright 2019, MDPI.

Among the MOF family, MOF-5 (also known as IRMOF-1) is one of the most well-studied examples, featuring a three-dimensional framework made from terephthalic acid and Zn4O metal clusters (Karimzadeh et al., 2019). MOF-5 is particularly noted for its open skeleton structure, controlled pore size and surface area, and high thermal stability, making it an attractive candidate for medical applications (Javanbakht et al., 2020). Chen et al. utilized direct addition synthesis to produce MOF-5 and solvent adsorption techniques to load ORI (ORI@MOF-5). The ORI@MOF-5 system demonstrated sustained release properties under various pH conditions, along with good biocompatibility and biodegradability. This sustained-release functionality helps to minimize drug toxicity and side effects, making it a promising candidate for anticancer therapy (Chen et al., 2019). Additionally, efforts have been made to modify MOFs with substituents to improve ORI delivery by personalizing DDS and tailoring the physicochemical properties of MOFs to meet specific drug-targeting needs (Cai et al., 2020) (Figure 7).

Sustained-release formulations remain a key focus in cancer drug research. Iron carboxylate MOFs, composed of iron ions and terephthalic acid, exhibit non-toxicity and biocompatibility. Leng et al. (2018) synthesized a flexible porous MOF as a carrier for ORI, confirming its high drug-loading capacity, favorable biocompatibility, and excellent sustained-release characteristics. These findings underscore the potential of MOF-based delivery systems to enhance drug loading efficiency and biocompatibility, making them promising candidates for the development of sustained-release agents to bolster the anticancer activity and therapeutic efficacy of ORI (Cai et al., 2022; Shen et al., 2024).

### 4.4 Microemulsions

Melf-microemulsifying drug delivery systems (SMEDDS) are isotropic mixtures composed of oil, surfactants, co-surfactants, and drug compounds (Dokania and Joshi, 2015). These systems enhance the solubility of poorly water-soluble drugs, thereby improving their absorption through oral administration (Kovačević et al., 2022).

**FIGURE 8:**
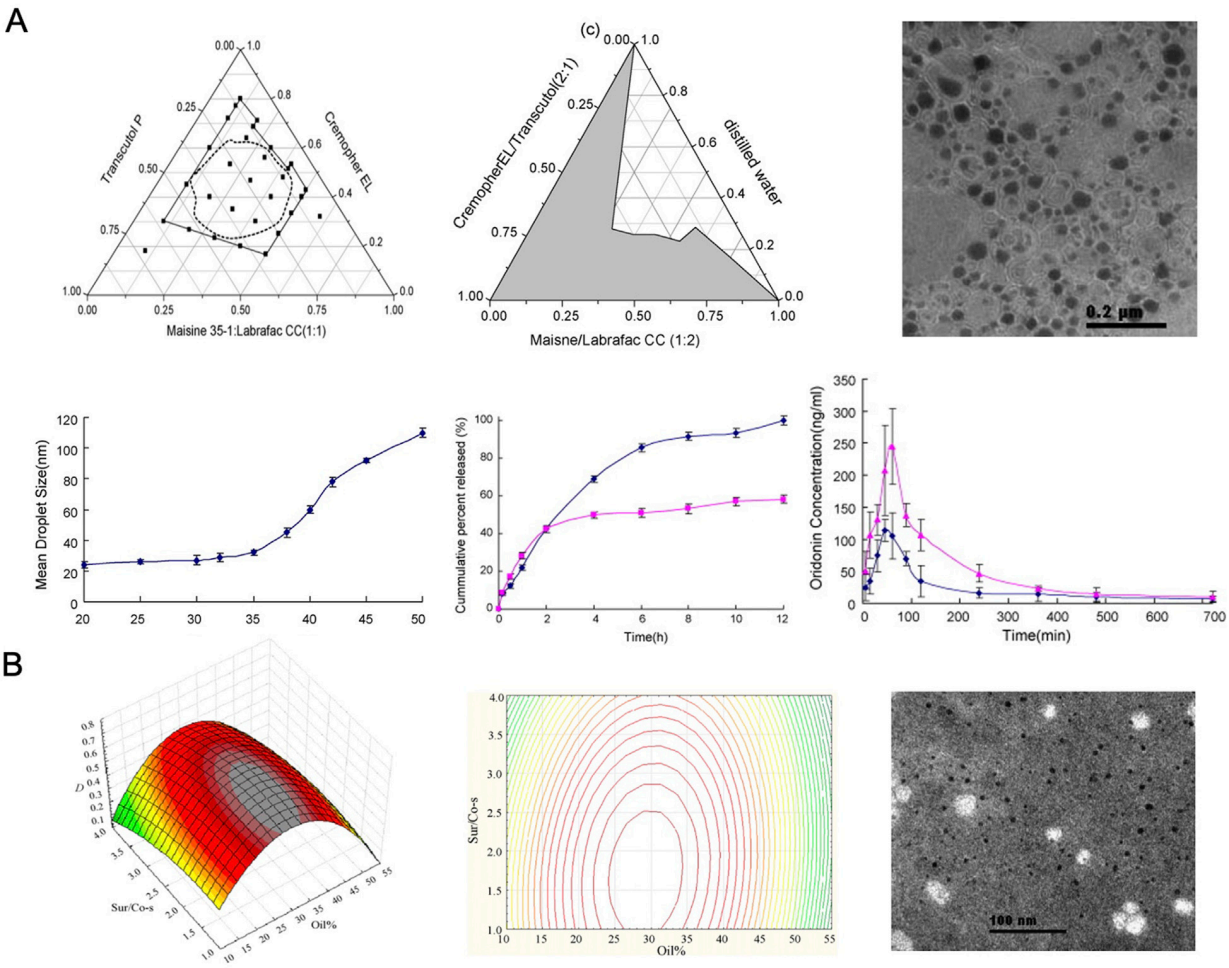
**(A)** Spherical microemulsion droplets. Reproduced with permission from Ref (Zhang et al., 2008). Copyright 2008, Elsevier. **(B)** Optimized SMEDDS formulation. Reproduced with permission from Ref (Liu et al., 2009). Copyright 2009, Elsevier.

Zhang et al. (2008) developed an ORI-based oral microemulsion using Maisine 35-1 and Labrafac CC, which, compared to a suspension, significantly enhanced the drug’s bioavailability, indicating its potential for oral use. The performance of SMEDDS is influenced by the ratio of its components and the drug concentration. To address this, Zhang et al. optimized the formulation by identifying the ideal ratio of microemulsifiers, leading to improved intestinal absorption and rapid drug release characteristics. Additionally, SMEDDS, as a lipid-based delivery system, facilitates partial absorption through the lymphatic pathway, which may bypass first-pass hepatic metabolism and further enhance bioavailability (Figure 8).

### 4.5 Nano suspension

Nano suspensions represent a carrier-free colloidal drug delivery system, consisting primarily of pure drug NPs smaller than 100 nm, stabilized with minimal surfactant (Kovalchuk et al., 2019). These systems offer versatility in administration, supporting oral, intravenous, and parenteral delivery routes (Gao et al., 2008). One example is the hydroxypropyl-β-cyclodextrin (HP-β-CD) inclusion complex of ORI, which was used to create nanosuspension solutions for oral administration. The inclusion of HP-β-CD significantly enhanced intestinal solubility, effective permeability, and bioavailability (Zhang et al., 2016).

**FIGURE 9:**
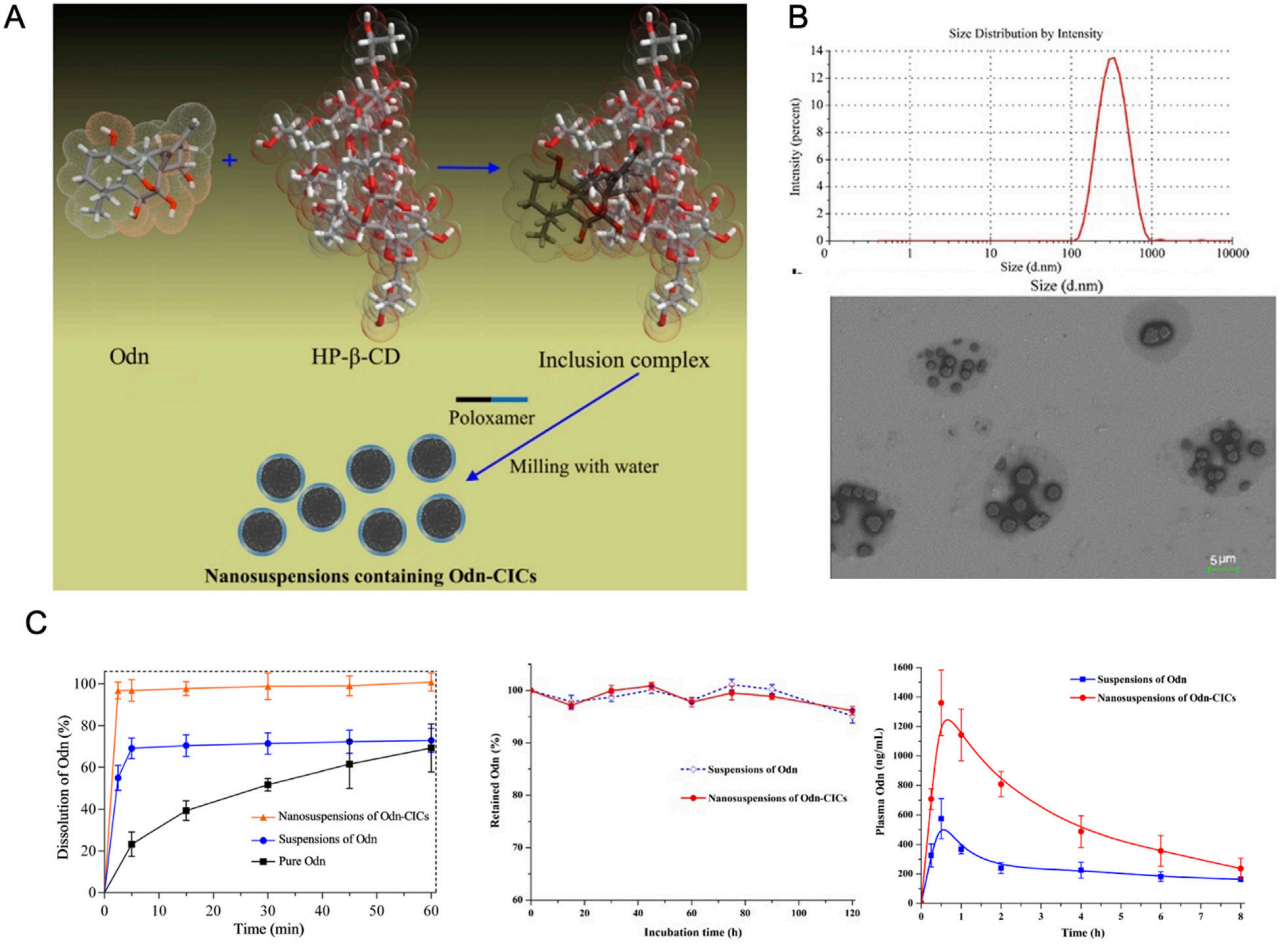
**(A)** Illustrations of the inclusion and preparation processes of ORI/HP-β-CD. **(B)** Characterization of HP-β-CD. **(C)** Pharmacokinetics of different drug delivery systems. Reproduced with permission from Ref (Zhang et al., 2016). Copyright 2010, Taylor & Francis.

Both *in vitro* and *in vivo* studies have demonstrated the superior antitumor effects of ORI formulated as a nanosuspension compared to its free drug form. *In vitro*, the nanosuspension displayed greater cytotoxicity and a higher rate of apoptosis induction. *In vivo* studies in mice showed that ORI nanosuspensions were better tolerated, offering enhanced anti-tumor efficacy with reduced toxicity (Lou et al., 2009; Feng et al., 2011). Specifically, ORI nanosuspension exhibited stronger cytotoxicity against human pancreatic cancer PANC-1 cells (Qi et al., 2012), and its formulation significantly boosted the anti-tumor activity in human breast cancer MCF-7 cells, increasing both cell cycle arrest and apoptosis (Feng et al., 2011) (Figure 9).

Further *in vitro* studies on human prostate cancer PC-3 cells revealed that ORI nanosuspensions provided more pronounced inhibition of cell proliferation, significantly enhancing growth inhibition and apoptosis induction (Zhang et al., 2010). Lou et al. (2011) confirmed that ORI nanosuspension also demonstrated superior antitumor activity in H22 tumor-bearing mice while reducing toxicity. Additionally, it has been shown that particle size significantly affects the pharmacokinetics and tissue distribution of ORI nanosuspensions following intravenous administration. Optimizing particle size and developing high-bioavailability oral formulations could make ORI nanosuspensions a more viable candidate for clinical use (Gao et al., 2007).

### 4.6 Protein-based nanocarriers

Among functional nanomaterials, proteins are considered highly effective drug carriers due to their excellent adsorption properties, non-toxicity, non-immunogenicity, and favorable *in vivo* stability. Bovine serum albumin (BSA) NPs, in particular, have emerged as a promising drug delivery platform (Wang and Zhang, 2018; He et al., 2023). These macromolecular proteins protect active drug substances from hydrolytic degradation, reduce cardiotoxicity, enhance water solubility and biocompatibility, prolong blood circulation time, and minimize toxic side effects (Teran-Saavedra et al., 2019; Wang and Zhang, 2018). A BSA-based ORI-loaded galactosylated BSA nanoparticle system (ORI-GB-NP) has been developed for the liver-targeted delivery of ORI (Li et al., 2013). Parenteral delivery experiments revealed that ORI-GB-NP increased plasma drug levels, extended circulation time, and enhanced liver delivery while reducing toxicity in the heart, lungs, and kidneys (Li et al., 2014).

**FIGURE 10:**
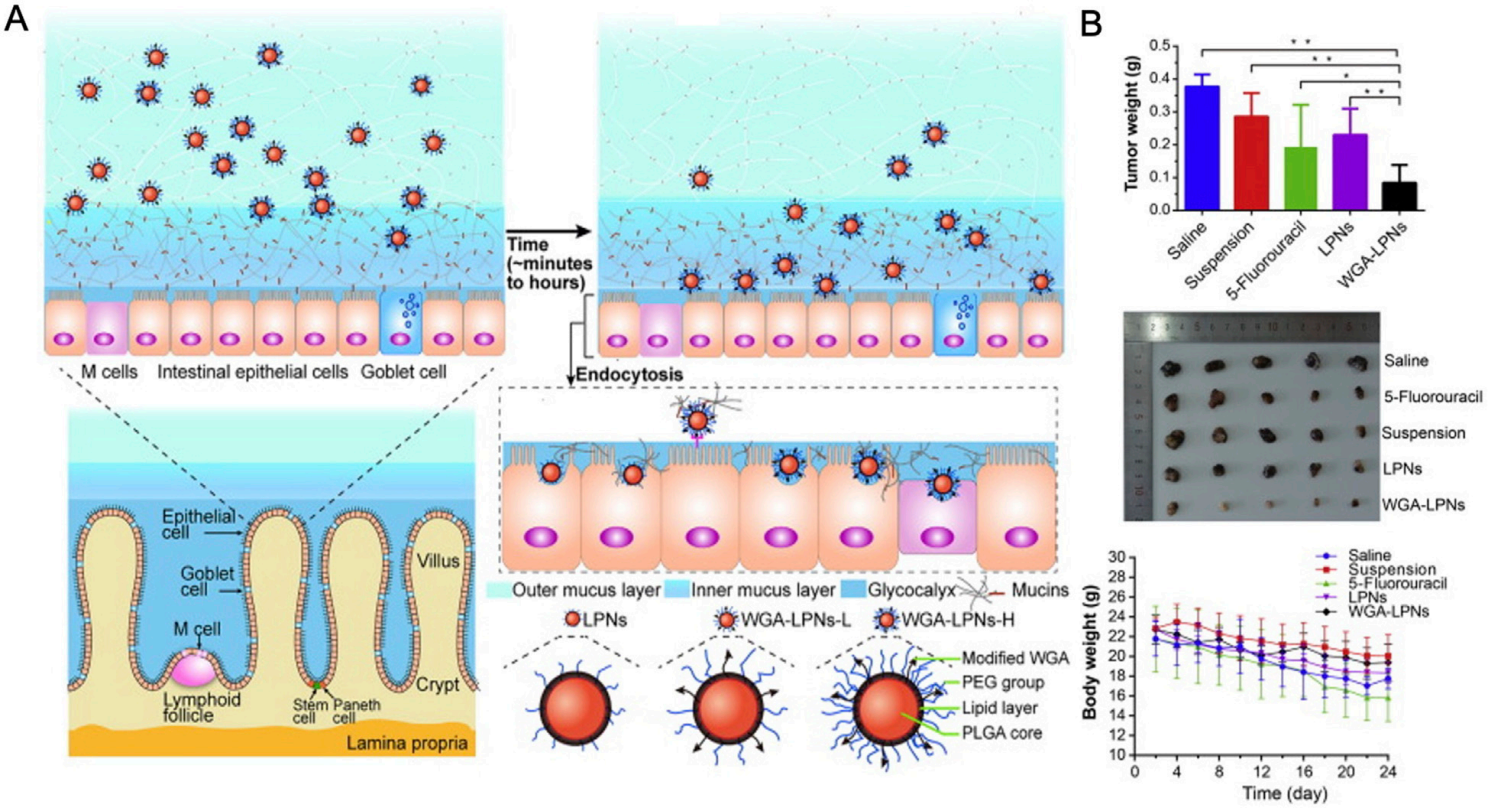
**(A)** Preparation and mechanism of WGA-LPNs. **(B)** Tumor inhibition effects after treatment in each group. Reproduced with permission from Ref (Liu et al., 2017). Copyright 2017, Elsevier.

Additionally, wheat germ lectin-modified lipid-polymer hybrid NPs (WGA-LPNs) possess receptor-mediated endocytosis and bioadhesive properties, promoting cellular uptake after oral administration (Liu et al., 2011). ORI-loaded WGA-LPNs exhibited significantly greater extracellular uptake and intestinal diffusion than the control group, leading to a 1.96-fold increase in oral bioavailability and pronounced induction of cell apoptosis (Figure 10) (Liu et al., 2017). NPs, due to their unique physical and chemical properties, inherently possess high surface free energy, which makes them unstable. However, stability can be achieved by binding NPs to biomolecules such as proteins, which helps reduce free surface energy. Albumin-based NPs have demonstrated the ability to enhance plasma drug concentration, improve solubility, achieve predictable biodistribution, ensure biocompatibility, and effectively bind to a range of therapeutic agents (Mariam et al., 2016).

### 4.7 Polymer NPs

Polymeric NPs offer chemical diversity and the potential for long-term and targeted drug delivery *in vivo* (Ferreira Soares et al., 2020). Sun et al. (2024) developed a liver-targeted ORI delivery system using Angelica sinensis polysaccharide (ASP) as the carrier and formulated ORI-loaded ASP-deoxycholic acid (DOCA) NPs (ORI/ASP-DOCA NPs). This system achieved high drug loading and utilized asialoglycoprotein receptor-mediated targeting in H22 tumor-bearing mice, resulting in significant anticancer effects while minimizing systemic toxicity (Sun et al., 2024).

**FIGURE 11:**
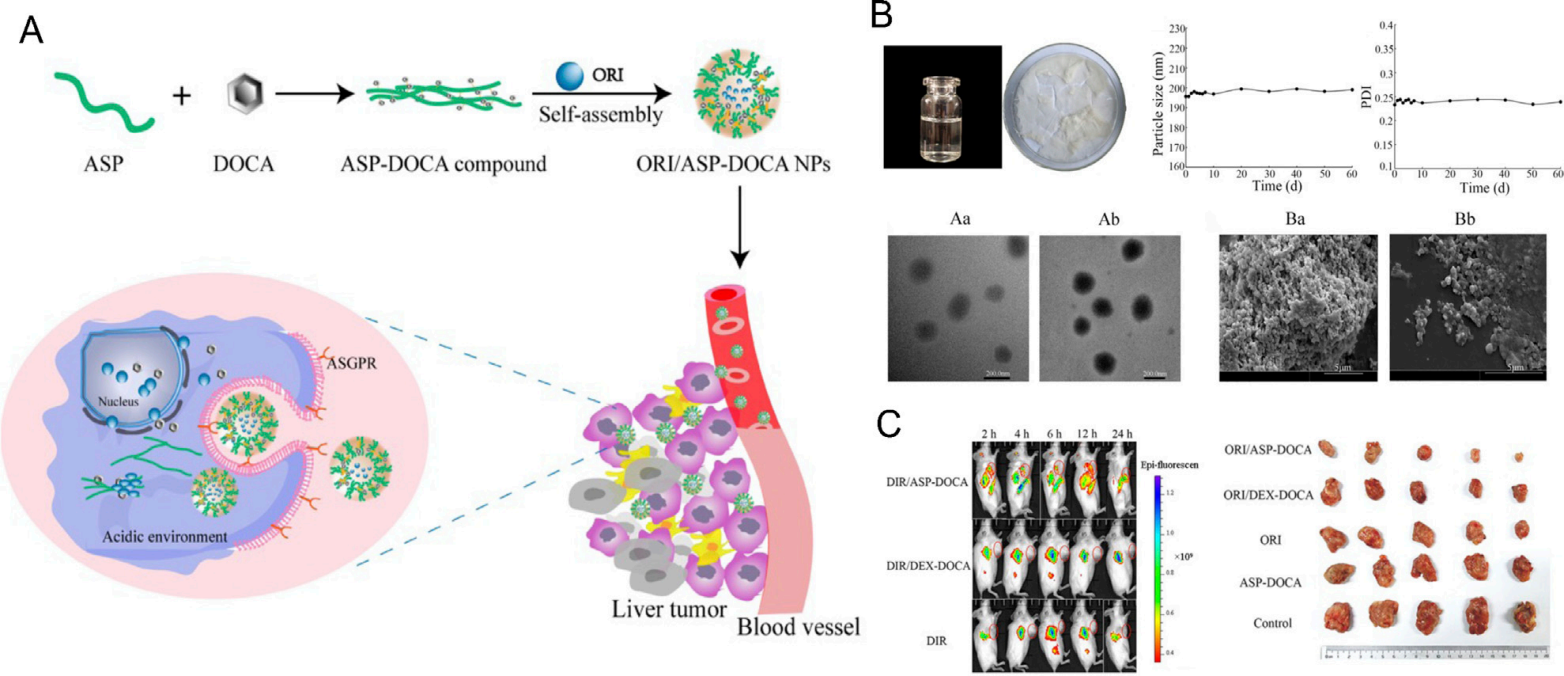
**(A)** Schematic design of ORI/ASP-DOCA NPs. **(B)** Characterization of ORI/ASP-DOCA NPs. **(C)** Tumor inhibition effects after treatment in each group. Reproduced with permission from Ref (Zhang et al., 2016). Copyright 2024, MDPI.

In another study, ORI-loaded random poly (D, L-lactic acid) NPs (ORI-PLA-NPs) were prepared via a modified spontaneous emulsification solvent diffusion method. The pharmacokinetics, tissue distribution, and anticancer activity of these NPs were evaluated in mice with H22-derived tumors. The results showed that ORI-PLA-RGD-NPs exhibited superior tumor targeting and anti-tumor efficacy compared to ORI-PLA-NPs or ORI solution, with a higher accumulation of ORI in tumor tissues (Xu et al., 2012) (Figure 11).

Zheng et al. (2012b) also developed galactose-chitosan-coated ORI-loaded NPs (ORI-GC-NP) for targeted cancer therapy. These NPs exhibited pH-dependent release behavior in acidic environments, enhancing ORI release specifically within the acidic tumor microenvironment. ORI-GC-NP demonstrated strong liver-targeting capabilities and prolonged plasma retention, making it a promising strategy for liver cancer treatment (Zheng et al., 2012b). Various polymers have been explored for developing ORI delivery systems, with some exhibiting excellent biocompatibility and tumor-targeting properties, while others have shown enhanced tumor-specific release, improved solubility, and extended circulation time. Polymeric NPs are proving to be an especially promising carrier for ORI.

## 5 Advantages of nano-delivery systems

### 5.1 Pharmacokinetic properties

The study of drug pharmacokinetics, encompassing absorption, distribution, metabolism, and excretion, plays a critical role in minimizing adverse reactions and determining appropriate dosages. It also aids in the design of dosage regimens and optimization of clinical efficacy (Glassman and Muzykantov, 2019).

Pharmacokinetic analysis has become a key component of both preclinical and clinical drug development (Miller et al., 2019). For example, in studies assessing ORI in rat plasma after intragastric administration, the liver was identified as the primary site of metabolism. Verapamil, known to inhibit P-glycoprotein (P-gp) activity, has been shown to significantly affect the pharmacokinetic profile of ORI, leading to increased peak plasma concentration and bioavailability by enhancing its absorption (Liu et al., 2019).

A notable case is the ORI/2-hydroxypropyl-β-cyclodextrin inclusion complex nanosuspension, which substantially improved relative bioavailability, highlighting the potential of nanosuspensions prepared from inclusion complexes for oral anticancer drugs. In another study, Li et al. (2011b) developed a solid dispersion using gas antisolvent technology, where ethanol served as the solvent, CO_2_ as the antisolvent, and polyvinylpyrrolidone K17 as the carrier matrix. This solid dispersion formulation resulted in a 26.4-fold increase in ORI bioavailability (Li et al., 2011b).

Lin et al. (2023) developed and validated a highly sensitive HPLC method with UV detection for quantifying ORI in rat plasma. ORI liposome administration led to a significant extension of the elimination half-life and an increase in the AUC, along with a reduction in the clearance rate (Lin et al., 2023). Nanocarriers play a vital role in improving drug bioavailability and prolonging half-life. Understanding the alterations in pharmacokinetics and conducting thorough pharmacokinetic studies are crucial for elucidating the biological functions and mechanisms of these carriers *in vivo*. Optimizing pharmacokinetic properties is essential for the successful application of nanomedicine in cancer therapy.

### 5.2 Enhanced tumor inhibition

The type and proportion of ligands on the surface of NPs, along with the particle size of the delivery system, can significantly influence the circulation, cellular uptake, and drug delivery profile of NPs. These factors play a key role in promoting cellular endocytosis and modulating the rate of drug delivery, which can impact the penetration and uptake of ORI within the body (Danaei et al., 2018).

A reduction in ORI’s bioavailability may limit its anticancer efficacy *in vivo*, as its poor solubility presents a major challenge in clinical applications (Fan et al., 2018). Therefore, enhancing ORI bioavailability, particularly for oral DDS, is critical, given the barriers posed by the gastrointestinal tract (Parodi et al., 2021). Studies have shown that PEG-modified nanocarriers can improve drug permeability, thus enhancing bioavailability and boosting the anticancer potency of drugs like ORI (Ren et al., 2021; Fang et al., 2016; Chai et al., 2019).

Particle size is another important factor, affecting both intravenous administration and oral absorption of ORI. While reducing particle size can improve tissue permeability, it may also compromise stability, necessitating careful selection of an appropriate particle size to optimize the delivery effect of each DDS (Danaei et al., 2018).

Long-circulating drug carriers are particularly advantageous in DDS, as prolonged circulation can increase drug accumulation at the target site, while overly rapid clearance may prevent drugs from achieving their full therapeutic potential (Ou et al., 2018). For instance, nanostructured lipid carriers loaded with ORI have demonstrated extended circulation times (Zheng et al., 2012a), and stealth liposomes have been shown to significantly lengthen blood circulation times in pharmacokinetic studies (Wang et al., 2009) (Li et al., 2014). Achieving the right balance between circulation time and stability is therefore essential for maximizing the therapeutic efficacy of ORI in cancer treatment.

### 5.3 Reduce systemic toxicity

A significant challenge in oncology is managing the adverse drug reactions (ADRs) associated with anticancer agents, which can severely impact patient safety and quality of life (Mhandire and Goey, 2022). Reducing drug toxicity while improving safety are critical factors for the successful development of anticancer therapies. The use of protein-based nanocarriers has proven effective in decreasing toxicity and the incidence of adverse effects linked to pharmaceutical agents. Protein modifications, in particular, can help mitigate ORI’s toxicity to organs such as the heart, lungs, and kidneys (Spada et al., 2021; Haddadzadegan et al., 2022). *In vitro* studies of ORI release have revealed a biphasic drug release profile, with an initial burst followed by sustained release.

Active targeting, which employs ligand-modified carriers to deliver drugs directly to specific targets, holds promise for enhancing drug accumulation at the site of action while reducing systemic toxicity, offering a substantial improvement over passive targeting (Tang et al., 2021; Bandyopadhyay et al., 2023). For instance, folate receptor (FR)-targeting ORI-loaded liposome microbubbles (F-LMB-ORI) demonstrated a higher binding affinity to HepG-2 cells, significantly improving drug efficacy (Wang et al., 2017).

In summary, the ORI delivery systems reviewed in this paper enhance anticancer activity by prolonging circulation time, improving tumor targeting, enhancing solubility and bioavailability, optimizing particle size, and reducing toxicity.

## 6 Market application and clinical trials

ORI has shown notable clinical applicability and the potential for further therapeutic development. Several ORI-based formulations have already received approval and are available for treating various inflammatory conditions, such as tonsillitis, pharyngitis, laryngitis, and stomatitis (Wu et al., 2024). For example, ORI tablets and ORI drop pills are recommended as adjuvant cancer therapies (Table 3). Hu et al. developed a series of enmein-type diterpenoid amino acid ester derivatives, including L-alanine-(14-ORI) trifluoroacetate, one of which (Compound 19) exhibited potent cytotoxicity against both HCC Bel-7402 and chronic myelogenous leukemia K562 cells, while demonstrating some selectivity (Hu et al., 2019). Hengrui Medicine Co., a Chinese pharmaceutical company, conducted the first human clinical trial (CTR20150246) of HAO472, an L-alanine-(14-ORI) ester trifluoroacetate, as a potential treatment for acute myelogenous leukemia (Hu et al., 2020). Additionally, Rabdosia rubescens Drops are currently being evaluated in a phase IV clinical study (ChiCTR1800015210) for their efficacy and safety in preventing and treating radiological oral mucositis in patients with nasopharyngeal carcinoma, as registered in the China Clinical Trials Registry (Table 4).

**TABLE 3:** Representative marketed drugs related to ORI.

Clinical drugs	Preparation method	Treatment	Administration	Approval no.
ORI tablets	Mixed compression method	Inflammation, cancer adjuvant therapy	Oral	Z41020014 Z43020508 Z41022300
ORI dripping pills	Mixed compression method	Inflammation, cancer adjuvant therapy	Oral	Z20150003
ORI syrup	Extraction and purification method	Tonsillitis, pharyngitis, laryngitis, and stomatitis	Oral	Z41020928 Z41022229 Z41022340 Z41021631
ORI capsule	Extraction and purification method	Tonsillitis, pharyngitis, laryngitis, and stomatitis	Oral	Z20060139 Z20080344

**TABLE 4:** Ongoing clinical trials related to ORI.

Clinical drugs	Preparation method	Treatment	Administration	Clinical trial no.	Phase
HAO472	Esterification reaction, removal of protective groups and salt formation	Acute myelogenous leukemia	Intravenous drip	CTR20150246	I
*Rubescens* dripping pills	The hot melt method of solid dispersion technology	Acute pharyngitis Acute tonsillitis	Oral	ChiCTR2300076500	IV
ORI tablets combined with cytarabine	Mixed compression method	Acute myeloid leukemia	Oral	ChiCTR2200059551	IV
*Rubescens* dripping pills	The hot melt method of solid dispersion technology	Radiation-induced oral mucositis	Oral	ChiCTR1800015210	IV
ORI tablets	Mixed compression method	β-thalassemia	Oral	ChiCTR-OOB-16007883	IV
*Rubescens* dripping pills	The hot melt method of solid dispersion technology	Coronary artery disease (CAD)	Oral	ClinicalTrials.gov IDNCT05130892	IV

At present, most ORI-based drug formulations available on the market consist of conventional tablets, drops, and capsules, while no ORI-based nanomedicines have been marketed to date. This suggests that ORI nanodrug development is still largely in the animal testing phase, indicating that widespread clinical application remains some distance away. Furthermore, most marketed drugs and clinical trials are based in China, the origin of traditional Chinese medicine. Expanding ORI’s therapeutic indications and promoting its global usage are critical areas for future research.

## 7 Conclusion and future considerations

The ideal DDS maintains drug concentrations within the therapeutic range or targets delivery specifically to affected organs and tissues while minimizing exposure to other regions, thereby reducing systemic toxicity. Despite ORI’s well-documented anticancer efficacy (Tarantino et al., 2023), its clinical use is limited by poor solubility and bioavailability (Unsoy and Gunduz, 2018). Overall, the continuous development of ORI-based DDSs provides promising avenues for cancer therapy, though certain limitations remain, accompanied by specific drawbacks and challenges. Some nanocarriers exhibit incomplete biodegradability, which may lead to liver or kidney toxicity, while the precise targets of some carriers remain unclear, and research on the accurate tissue and organ distribution of ORI is still limited (Ding et al., 2016).

Specifically, ORI liposomal DDSs may degrade *in vivo* due to lipid oxidation or hydrolysis, leading to premature drug release and thus compromising therapeutic efficacy. Their stability issues also pose challenges for storage and transportation (Liu et al., 2024b). ORI micellar DDSs, often composed of polymers and surfactants, are not sufficiently stable under physiological conditions. In particular, micellar particles may disassemble in the bloodstream, resulting in premature drug release and reduced targeting efficiency. Several MOF DDSs have been employed in previous studies on ORI nanotechnology, but these have primarily served as frameworks for ORI delivery to enhance its stability, with the MOFs themselves did not exhibit significant antitumor effects (Chen et al., 2019). ORI suspensions may aggregate during storage or transport, leading to increased particle size, which can affect the uniformity and stability of drug distribution. Proteins used to deliver ORI, as natural biomolecules, are susceptible to enzymatic degradation, thermal denaturation, or alterations in pH within the body, potentially causing rapid DDS degradation and premature drug release, thereby diminishing therapeutic efficacy (Teran-Saavedra et al., 2019). While the tumor microenvironment is generally acidic, not all tumor sites maintain a consistently low pH. Even if certain ORI DDSs show favorable release profiles *in vitro* under acidic conditions, it cannot be guaranteed that they will function effectively in all tumors, especially in those with high heterogeneity (Shen et al., 2024).Although targeted ORI DDSs demonstrate high selectivity, in the complex tumor microenvironment, targeting molecules may sometimes fail to bind precisely to the target receptors, particularly in tumors with heterogeneous expression of these molecules (Sun et al., 2024).

While several ORI-based drugs have been introduced to the market, their range of indications is relatively narrow, focusing primarily on common inflammatory diseases and serving as adjunctive therapies for cancer (Liu et al., 2020; Yan et al., 2020). Current formulations, such as tablets and capsules, are conventional and lack innovative delivery methods. This limited variety in formulation design not only constrains the broader clinical applicability of ORI but may also impact its bioavailability and patient compliance. The absence of diverse and advanced DDSs restricts ORI’s therapeutic potential, particularly when addressing complex diseases or conditions involving multiple complications. Moreover, ORI-based nano-drugs are still in the experimental stage and have not yet been commercialized, preventing the full clinical translation of their potential therapeutic benefits (Hu et al., 2020). Although laboratory studies indicate that ORI nano-drugs exhibit excellent efficacy and targeting capabilities, the lack of large-scale clinical trials leaves their safety and effectiveness unproven.

Future research on ORI should emphasize several critical aspects to advance its role in drug development. Although nano-drug formulations offer significant potential, addressing toxicity and safety remains paramount. The use of biocompatible delivery materials, such as polymer NPs and liposomes, is recommended to mitigate potential side effects. Current studies largely focus on ORI, yet Rabdosia rubescens contains multiple active constituents. A broader investigation into these components and their synergistic effects is warranted (Liu et al., 2020; Yan et al., 2020). Combining ORI with other extracts to develop composite nano-drug systems could further enhance therapeutic efficacy and expand its clinical applications. Additionally, beyond cancer treatment, the potential of ORI in managing cancer-related symptoms like pain, nausea, and fatigue should be explored. Conducting clinical studies to investigate ORI’s efficacy in alleviating these symptoms could expand its systemic utility and increase its market potential.

With the rapid advancements in nanotechnology, researchers should explore and develop more sophisticated and rational novel nanodrug delivery systems, such as targeted delivery technologies that promote the accumulation of ORI nano-drugs in specific tissues. This would improve therapeutic outcomes. Furthermore, smart drug delivery systems capable of precise, controlled drug release may unlock new possibilities for ORI’s clinical use. Certain noble metals (Au NPs, Ag NPs, and Pt NPs) (Qin et al., 2022), and non-noble metals (Cu NPs and Mg NPs) exhibit strong antitumor effects (Zhao et al., 2022a), functioning both as drug carriers and tumor inhibitors (Chen et al., 2024b). Their combination with ORI DDS may lead to better efficacy and present the possibility of combination with photothermal therapy. Multi-strategy combination therapy is an inevitable direction for future clinical practice (Chen et al., 2021; Zhang et al., 2024), and the existing sonodynamic DDS and chemodynamic DDS are far from sufficient to meet clinical needs. There is a necessity to further improve carrier design and adopt rational research strategies, aiming to develop more DDS based on PTT (Zhao et al., 2022b), electrodynamic therapy (Qiao et al., 2022), radiodynamic therapy (Matsuyama et al., 2022), magnetic hyperthermia (Vangijzegem et al., 2023), immunotherapy (Lu et al., 2024), and gene nanotherapy (Liu et al., 2024c). Given that ORI research spans multiple disciplines, including pharmacology, materials science, and clinical medicine, interdisciplinary collaboration is essential for advancing ORI-based drug development. Collaborations with biomedical engineers to design innovative nano-carriers and partnerships with clinical researchers for multi-center trials could accelerate progress in this area.

While ORI faces certain challenges in drug development, its therapeutic potential remains significant. Addressing toxicity, safety, active component analysis, expanding indications, and fostering technological innovation will likely solidify ORI’s role in modern medicine. As research progresses and technology evolves, ORI-based nano-drugs could offer substantial benefits to a broader range of patients, contributing to the modernization of traditional Chinese medicine.
